# Preparing for Patient Care: A Pre-clerkship Curricular Thread to Improve Learners’ Knowledge, Attitudes, and Confidence Treating Patients at Risk for Suicide

**DOI:** 10.1007/s40670-024-02186-7

**Published:** 2024-10-09

**Authors:** Kirsten A. Porter-Stransky, Angela C. Davio, Perry Westerman

**Affiliations:** 1 Department of Biomedical Sciences, University of South Carolina School of Medicine Greenville, 607 Grove Rd, Greenville, SC 29605 USA; 2 Department of Biomedical Sciences, Western Michigan University Homer Stryker M.D. School of Medicine, Kalamazoo, MI USA; 3 Department of Psychiatry, Western Michigan University Homer Stryker M.D. School of Medicine, Kalamazoo, MI USA

**Keywords:** Mental health, Stigma, Curricular thread, Spiral curriculum, Active learning

## Abstract

Suicide is a leading cause of death. Unfortunately, stigma still surrounds depression and mental health treatment. Many health care providers are uncomfortable broaching the topic with patients. There is an urgent need to better equip future physicians to compassionately identify and treat patients at risk for suicide. To address this problem, we created a novel suicide prevention spiral curricular thread for advanced pre-clerkship medical students. Mixed methods surveys were administered before and after the curriculum. Following completion of the curriculum, learners’ confidence identifying and treating patients at risk of suicide significantly increased. Attitudes including professional confidence, therapeutic optimism, and generalist perspective measured by the Revised Depression Attitudes Questionnaire also increased, indicating reduced stigmatizing attitudes and increased knowledge and confidence. Participants reported that engaging in the standardized patient encounter was the most impactful event, although the prior events provided necessary foundational training. This study demonstrates the feasibility and effectiveness of a curricular thread approach during the pre-clerkship phase for training medical students in suicide prevention to prepare them for patient care. Other medical schools can use this framework to design and integrate suicide prevention training into their own curricula.

## Introduction

Depression is a common psychiatric condition, and over 700,000 deaths by suicide occur worldwide annually [1]. In the USA, deaths from suicide are approaching 50,000 annually, with 12.3 million adults seriously considering suicide in just 1 year [2, 3]. Studies in the USA reveal that nearly half of those who died by suicide were seen in the health care system within the 4 weeks preceding their death, but the majority of these patients did not have a mental health diagnosis [4, 5]. Patients who die by suicide are more likely to have been treated by a primary care physician than a psychiatrist [6], indicating that training in suicide prevention should not be reserved for psychiatrists but should be accessible for all physicians, especially those in primary care. Unfortunately, depression and suicide remain stigmatized, creating barriers for both patients and health care providers to discuss it [7, 8]. Indeed, gaps in training about suicide prevention remain for many health care providers, and there is a need for implementation of evidence-based practices, such as the Zero Suicide Model [9]. Together, these findings indicate that the lack of provider comfort and competence is serious and pervasive.

Future physicians need better training for identifying and intervening with patients at risk of suicide. Guidelines on suicide prevention training from accrediting bodies are broad, leaving substantial flexibility to medical schools and residency programs in designing their objectives. For example, the word “suicide” is found neither in the Accreditation Council for Graduate Medical Education Program Requirements for Graduate Medical Education in Family Medicine nor in Internal Medicine [10, 11]. The same is true for the Liaison Committee on Medical Education guidelines, which emphasize the faculty of medical schools to define their own learning objectives, required clinical experiences, and competencies [12], providing opportunities for medical schools to customize such training for their learners. Some professional organizations, such as the Society of Teachers of Family Medicine, have a recommended clerkship curriculum that includes suicide risk assessment [13]; however, from our conversations with medical educators in this field, there is much room for improvement in depression and suicide prevention training in many programs. Indeed, previous work has highlighted the need for improved suicide prevention curricula in undergraduate medical education [14]. While teaching about suicide prevention is a necessary step, it alone is insufficient: examining medical students’ attitudes toward suicide and working to improve these attitudes is a critical component of training to promote compassionate and competent care [15].

The present study sought to fill this gap by developing, implementing, and evaluating a curricular thread on suicide prevention for pre-clerkship medical students. This curriculum was conceptualized from Kern’s six-step approach for curriculum development [16] and spiral curriculum [17] frameworks. An interdisciplinary team was formed with members from the disciplines of psychiatry, family medicine, psychology, neuroscience, wellness, and student life. This team consulted key stakeholders including administrators, faculty members, and students. The team discussed a variety of possibilities ranging from utilizing external programs to creating our own curriculum, dedicating a day to the topic versus weaving the topic throughout a curriculum, placing the content in a “doctoring” course versus biomedical science course, and positioning the content early versus later in the pre-clerkship curriculum. After evaluating the options, the team agreed for the course directors to create a suicide prevention thread (Table 1) integrated into a behavioral health course, the penultimate biomedical science course before clerkships. This later portion of the pre-clerkship phase specifically was chosen to build upon the foundational clinical skills training during the pre-clerkship phase and to equip students with necessary knowledge and skills for suicide prevention before they begin seeing patients in the clerkship phase of their medical training.

**Table 1:** Suicide prevention curricular thread. The curriculum included the following events weaved throughout a 4-week pre-clerkship behavioral health course. For additional information, readers are welcome to email the corresponding author

Teaching modality	Description of content
Asynchronous guided independent learning (1 h)	An online learning module developed by faculty introducing: • Epidemiology of suicide • Neurobiological and environmental factors contributing to suicide risk • Precipitating events and red flags for suicide risk • Screening for suicide, including the importance of phrasing, tone, and body language • Interventions for suicide ideation • Developing safety plans • Cultural and religious considerations • Crisis intervention resources for both patients and medical students
Lectures (1–1.5 h each)	Teaching about depression and suicide were included in the following lectures: • Depressive and bipolar disorders • Mood disorders: neurobiology and pharmacology • Medication-induced adverse effects (suicide risk and anti-depressants)
Case-based learning (CBL; 1.5 h) and team-based learning (TBLs; 3 h each; students in groups of 6–7; 2–3 faculty facilitators)	Application to cases • CBL on suicidality and violence, focusing on an elderly patient • TBL on anxiety and depression, focusing on a young adult patient • TBL on substance use and mood disorders, focusing on a patient experiencing homelessness
Suicide prevention tutorial (1 h for the students; ran in waves of 6 students at a time; could vary based on simulation center rooms and availability of standardized patients and faculty; ideally, need a 1:1:1 student/instructor/ standardized patient ratio but can run in waves or across days)	Standardized-patient encounter in the simulation center • Each medical student conducted a one-on-one 20-min interview of a standardized patient presenting in a primary care setting with headache and insomnia (due to depression) and suicidal ideation; the medical student was expected to screen for depression and suicidal ideation and begin developing a treatment plan • Following the role-playing interview, the standardized patient gave 5 min of feedback to the medical student about the learner’s communication skills • A psychiatry faculty member or senior resident observed the encounter and provided up to 30 min of individualized formative feedback; they discussed strengths, opportunities for improvement, how the experience impacted the learner, and anything else that the learner wanted to discuss (personally or professionally) following the experience

The suicide prevention thread was designed through a spiral curriculum framework, whereby topics were revisited with increasing levels of difficulty so that new learning would build upon previous learning to increase students’ competence [17]. Earlier events focused on foundational knowledge and understanding (lower levels of Bloom’s taxonomy), whereas later events required case application, analysis, patient evaluation, and creation of a treatment plan (higher levels of Bloom’s taxonomy). The first event was an asynchronous guided independent learning module so that learners could complete it at their own pace and in the location of their choice; this was purposeful because the topics of depression and suicide can elicit strong emotions, especially in individuals with personal or family psychiatric histories. Building upon foundational mental health and suicide prevention training (delivered through the asynchronous guided independent learning module and lectures), students applied the concepts in case- and team-based learning events. The curricular thread culminated with learners combining their aforementioned training on depression and suicide prevention to interview and treat a standardized patient in the simulation center (Table 1). The goals of this curricular thread were to equip learners with clinically relevant knowledge, skills, and attitudes to increase their confidence identifying and treating patients at risk for suicide before beginning clerkships.

## Materials and Methods

### Participants

To assess the impact of the new curricular thread on students’ knowledge of, attitudes about, and confidence in treating depression and suicide, mixed methods pre- and post-course surveys were distributed at the beginning and end of the course for 2 years. All second-year medical students enrolled in Behavioral Medicine, the penultimate pre-clerkship course at a private midwestern medical school in the USA, experienced the new suicide prevention thread and were invited to participate in the study. Survey participation was voluntary and anonymous, such that the course directors and researchers do not know which students participated and which did not. This study was deemed exempt under 45 CFR 46.101(b) by the Western Michigan University Homer Stryker M.D. School of Medicine Institutional Review Board and was conducted in accordance with their guidelines.

### Surveys

The previously validated 22-item Revised Depression Attitudes Questionnaire (R-DAQ) was used to assess learners’ attitudes about depression and suicide [18]. The questionnaire included three factors: professional confidence in depression care, therapeutic optimism about depression, and generalist perspective about depression occurrence, recognition, and management. Two survey items from Oordt et al. [19] were used to further probe participants’ confidence in assessing and treating suicide. All Likert-style questions were given on a 7-point scale to allow for both consistency throughout the survey and greater discrimination within half of the scale, because as medical students, participants were expected to have more positive attitudes and confidence at baseline than the general population. The R-DAQ originally was reported using a 5-point, rather than 7-point, Likert scale. Therefore, we ran a reliability statistic on the R-DAQ questions to ensure that this change did not invalidate the survey. The R-DAQ scale on the post survey had a Cronbach’s alpha value of 0.85, nearly identical to the 0.84 reported in the original article [18], demonstrating internal consistency of the R-DAQ with a 7-point scale. Surveys and data were stored in REDCap, a secure web application for building and managing online surveys. Participants generated their own unique 8-digit code that they typed on the pre and post surveys so that we could match pre and post results without compromising anonymity. Having a medical student on the research team who helped compile the survey questions provided important insight into how the questions, survey length, and timing would be perceived by learners.

### Analysis

Quantitative data were analyzed in GraphPad Prism version 10 (GraphPad Software, San Diego, CA) and SPSS version 29 (IBM Corporation, Armonk, NY). Two-tailed, paired *t*-tests were used to determine changes between the pre and post surveys in confidence in identifying and treating patients at risk for suicide. Repeated measures ANOVA was used to identify changes in R-DAQ scores, with Bonferroni post hoc tests to examine differences on the three factors of the R-DAQ. An alpha level of 0.05 was used to determine statistical significance. Data from both years were included in the analyses reported below. We also examined data from the 2 years separately and found the same results (not shown). Directed content analysis (performed by ACD and KAPS) was used to analyze the qualitative data about factors influencing students’ views.

## Results

This curriculum has successfully been implemented for 2 years (2022 (83 students) and 2023 (81 students)), and data were collected from both classes. Each year, over half the class participated in at least one survey, with 30% (25 students) year 1 and 36% (29 students) year 2 completing both pre and post surveys. In the pre survey of both years combined, 81% of respondents (65) reported receiving no formal training on suicide prevention prior to this course.

**Fig. 1:**
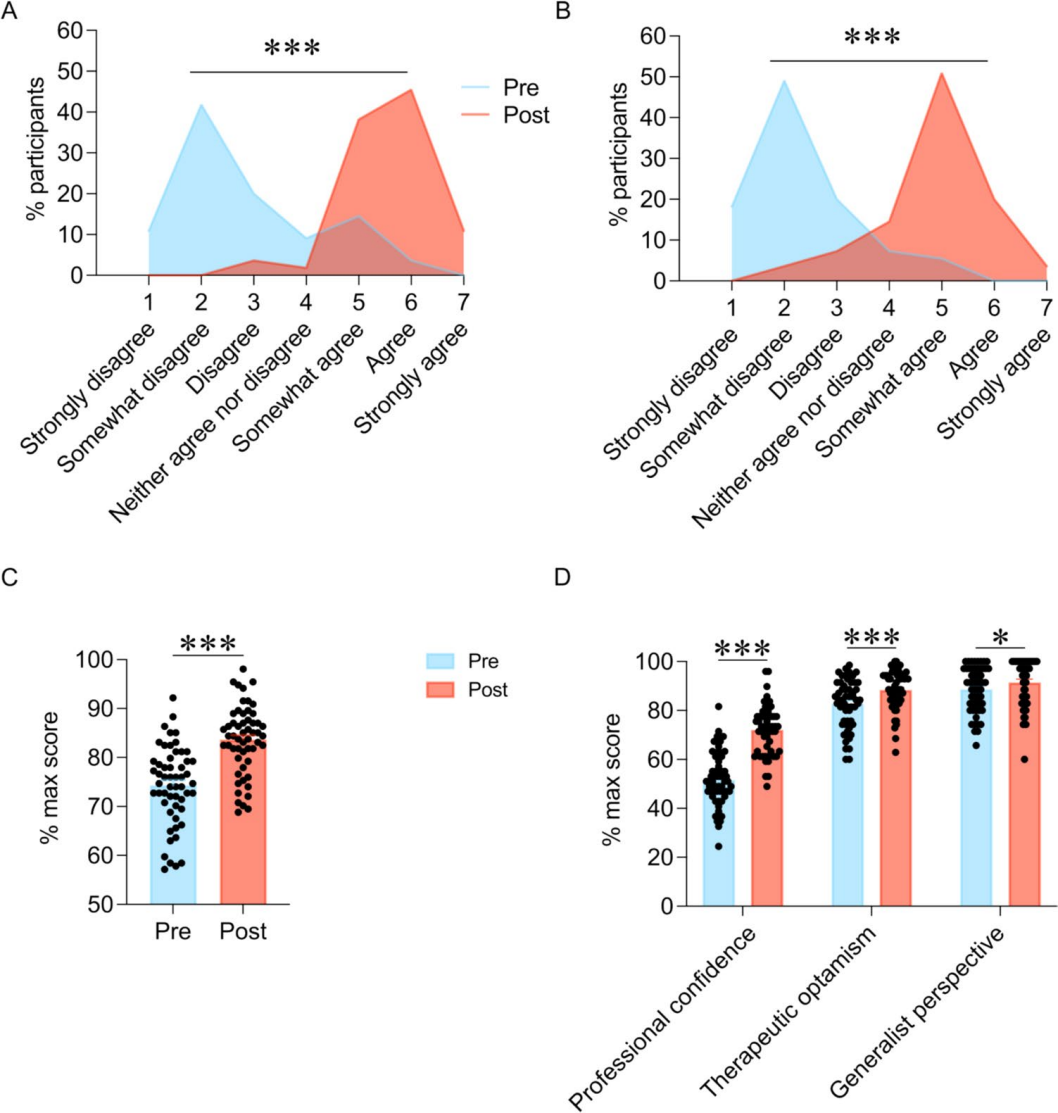
Impact of the suicide prevention thread on learners’ confidence and Revised Depression Attitude Questionnaire (R-DAQ) scores. **A**, **B** Participants’ self-ratings of their confidence to assess (**A**; “I am confident in my ability to successfully assess suicidal patients”) and treat (**B**; “I am confident in my ability to successfully treat suicidal patients”) those at risk for suicide increased following completion of the curriculum. **C–D** Participants’ attitudes about depression and suicide as measured by their overall Revised Depression Attitude Questionnaire (R-DAQ) scores (**A**) and their scores on the factors within the R-DAQ (**B**) improved after completing the suicide prevention thread. ***p < 0.0001, *p < 0.05, within-subjects comparisons of *n* = 54 second-year medical students

At the start of the course, most participants rated themselves low in confidence to successfully assess and treat patients at risk for suicide. However, by the end of the course, most students reported higher confidence in their ability to assess and treat patients at risk of suicide. Following completion of the new curriculum, there was a statistically significant increase in learners’ confidence in assessing (Fig. 1A; *t*_(53)_ = 15.06, *p* < 0.0001) and treating (Fig. 1B; *t*_(53)_ = 15.22, *p* < 0.0001) suicidal patients.

Additionally, students’ attitudes about depression as measured by the R-DAQ significantly improved following completion of the curriculum (Fig. 1C–D; within-subjects analysis: main effect of time, *F*_(1,53)_ = 187.22, *p* < 0.0001; interaction, *F*_(2,101)_ = 73.50,* p* < 0.0001). Post hoc analyses (Fig. 1D) revealed significant increases in professional confidence in depression care (*p* < 0.0001), therapeutic optimism about depression (*p* < 0.0001), and generalist perspective about depression occurrence, recognition, and management (*p* = 0.047). These results support the effectiveness of this novel curricular thread in improving learners’ confidence and knowledge as well as decreasing stigmatizing attitudes related to patient care for depression and suicide.

**Fig. 2:**
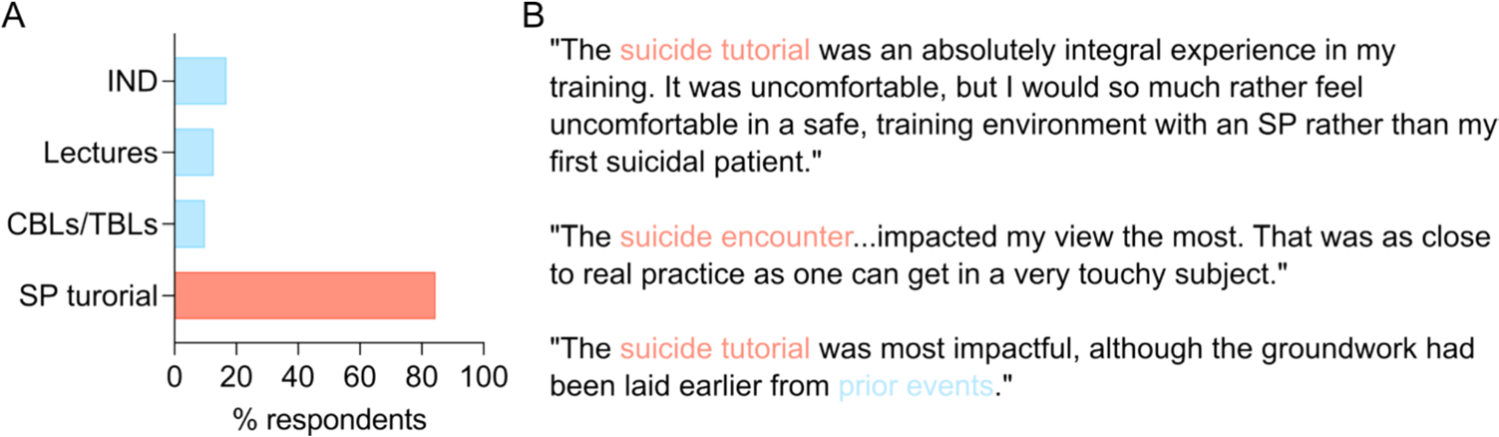
Components of the suicide prevention curriculum that students expressed as most impacting their views on depression and suicide.** A** Directed content analysis revealed that learners cited the guided independent learning event (IND), lectures, case-based learning (CBLs) and team-based learning (TBLs) events, and the suicide prevention tutorial with standardized patients (SP tutorial) in the simulation center as substantially impacting their views. **B** Representative quotations from participants explaining their thoughts on what impacted their views the most. *n* = 68 second-year medical students who wrote on the post-curriculum survey that a component of the suicide prevention curriculum impacted their views on depression and suicide

Seventy-one respondents gave one or more responses to the question, “What learning events or experiences have most impacted your views on depression and suicide?” on the post survey. Only six students (8%) mentioned non-curricular factors including personal experiences, family, or prior work as impacting their view. Ninety-five percent of respondents (68 students) cited at least one curricular event within the suicide-prevention thread. Each component of the suicide prevention curricular thread was mentioned as being impactful (Fig. 2A), with the overwhelming majority of participants citing the suicide prevention standardized patient tutorial as most impactful (Fig. 2A–B). Although this standardized patient encounter was most frequently referenced as being impactful, the other events importantly laid the groundwork.

## Discussion

The present study designed, implemented, and evaluated a suicide prevention curricular thread for pre-clerkship medical students. Although nearly all our medical students had clinical experience before matriculating to medical school, over 80% of respondents reported receiving no formal training in suicide prevention prior to this course, highlighting the need for such training. Quantitative and qualitative results demonstrate the effectiveness of this spiral curricular thread approach. Significant increases on each subscale of the R-DAQ support the interpretation that the curricular thread improved attitudes, confidence, and general knowledge about suicide and depression. Although most participants noted the standardized patient suicide prevention tutorial as the most impactful event, they recognized that they would not have been able to successfully complete it without the learning that occurred in prior events within the thread. This study demonstrates the feasibility and impact of longitudinal suicide prevention instruction before students are immersed in the clinic.

Strengths of this study include having study participation be optional and completely anonymous, collecting pre and post surveys for two cohorts, the use of previously published survey questions, the inclusion of both quantitative and qualitative data, and having a diverse research teaming including faculty members in behavioral neuroscience and psychiatry as well as a medical student. Limitations of the study include that it was conducted at one medical school, during one course, and we have yet to document the impact of the training on these learners’ future behaviors in clinic (Kirkpatrick Level 3) and their future patients’ outcomes (Kirkpatrick Level 4). The present study went beyond Kirkpatrick Level 1 (learners’ reactions) to meet Kirkpatrick Level 2 (learning, specifically acquiring “intended knowledge, skills, attitude, confidence, and commitment”) [20]. We expanded the response scale from 5 to 7 points; although this was a change to the previously validated R-DAQ, the Cronbach’s alpha value with this scale range in the present study was nearly identical. To minimize further changes to previously published surveys, we retained the phraseology of survey questions; however, as the culture of medicine changes, we hope that the terminology of new surveys will continue to evolve, such as using person-first language.

Strengths of the curricular thread approach include customization and integration of content for a school’s unique curriculum and learners, revisiting the material with increasing depth in a spiral curricular thread, completing this foundational training before patient care begins in clerkships, and discussing mental health resources for medical students and their future patients. Challenges include time, effort, and number of faculty needed to implement the curriculum. However, once the thread is established the first year, updating events subsequent years is relatively minor. Team-based learning and case-based learning allow for small group active learning with few instructors. The standardized patient suicide prevention tutorial was the most resource-intensive and impactful event. Including senior psychiatry residents as instructors helped ease the load of faculty and provided a new teaching opportunity for residents. Finally, expanding the duration of the thread (over multiple courses) is appealing; however, educational and practical considerations led us to weaving the thread throughout one course. Having this clinically relevant content later in the pre-clerkship phase was thought to be advantageous to help students scaffold this content upon the foundational clinical skills training that they acquired throughout the pre-clerkship phase. Additionally, the nature of this topic fit best within this mental health-focused behavioral health course, and this course is where the thread designers had power to most influence the curriculum.

This project adds to the medical education literature in providing an evidence-based approach for increasing future physicians’ confidence assessing suicide risk [21]. Our results are consistent with that of Desai et al. [14]; despite differences in location, training activities, and length of the curriculum, both studies support longitudinal training (days to weeks) on suicide prevention to improve medical students’ skills, knowledge, and attitudes. The consistency of findings (theirs in India and ours in the USA) suggest that this approach could be used worldwide; further research for implementing such training within different cultures would be beneficial. We posit that suicide prevention curricula across health professions education do not need to be identical; rather, the power is in the approach. Designing clinically and scientifically valid learning events tailored to one’s students and local needs, implementing them in a thread-like design, and having events build in complexity, requiring higher order application and analysis is an effective formula for improving learners’ confidence, skills, and attitudes for treating patients at risk for suicide.

## Conclusions

This project provides a framework for other medical schools to create and implement suicide prevention training as a curricular thread for their medical learners in an evidence-based way. Implementing mental health and suicide prevention training prior to clerkships provides medical students with needed skills, knowledge, and attitudes for their future patients. Follow-up research should examine the impact of such training on outcomes of trainees’ future patients. We hope that other programs will be inspired to dedicate the time to train all future physicians to compassionately and competently treat patients with depression or suicidal ideation.

## Data Availability

Data available upon reasonable request; contact the corresponding author.
